# Optical superhigh magnification dermatoscopy versus reflectance confocal microscopy: A practical approach to *in vivo* vascular biology imaging

**DOI:** 10.1016/j.jdcr.2025.07.031

**Published:** 2025-08-30

**Authors:** Luísa Polo-Silveira, Ashfaq Marghoob, Kivanc Kose, Miguel Cordova, Aditi Sahu

**Affiliations:** Dermatology Service, Department of Medicine, Memorial Sloan Kettering Cancer Center, New York, New York

**Keywords:** clinical research, dermatoscopy, oncology, optical super-high magnification dermatoscopy, tumor biology

## Clinical presentation

A patient presented with a basal cell carcinoma. Standard dermatoscopy highlighted arborizing vessels, but its insufficient cellular resolution limits evaluation of vascular dynamics.^1^ Although reflectance confocal microscopy (RCM) can provide *in vivo* cellular-level imaging, its grayscale images reduce cellular specificity and its high cost, complexity, and time requirements often hinder clinical accessibility.

## Dermatoscopic appearance

Optical superhigh magnification dermatoscopy (OSHMD) enables *in vivo* cellular-level imaging at up to 400× magnification, providing rapid, high-resolution, color-enhanced images. Its video capability supports real-time observation of biological processes, such as RCM but with added color contrast. RCM studies show that dynamic vascular events—such as tumor angiogenesis and leukocyte trafficking—within the tumor immune microenvironment influence antitumor immunity and treatment response.^2^ Although RCM offers high diagnostic precision, OSHMD presents a user-friendly alternative for vascular dynamics insights. Video 1 (available via Mendeley at https://data.mendeley.com/datasets/bjkwtvmdy7/1) demonstrates OSHMD imaging (FotoFinder D-Scope III; resolution 0.7 μm/px, field of view 1.35 × 0.76 mm, magnification 257×) showing blood trafficking within the lesion, compared with an RCM video of the same region (VivaScope 1500; resolution 0.7 μm/px, field of view 0.5 × 0.5 mm, 30–40 μm depth from the corneum).

Key message

**Fig 1:**
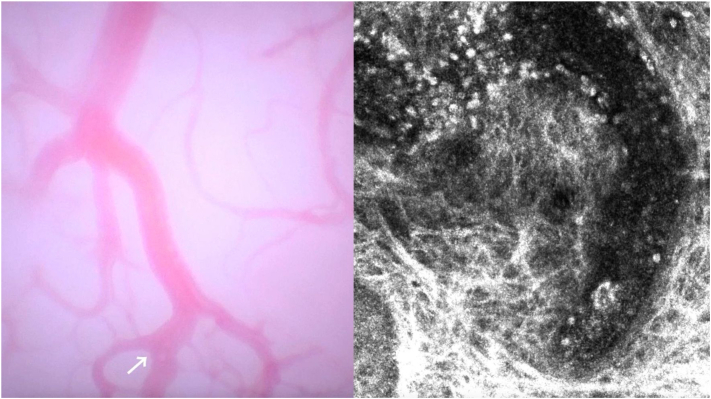
Frames from videos comparing blood trafficking using OSHMD and RCM in a basal cell carcinoma. Both methods show leucocytes traveling through blood vessels (*arrow*).

Both modalities reveal leukocytes circulating within tumor vessels (Fig 1), but OSHMDs color contrast adds value by distinguishing blood flow dynamics—erythrocyte-rich regions and leukocyte movement as white circular cells—despite lower resolution. OSHMDs limitations include operator dependency, potential artifacts, and limited literature support. Given these trade-offs, the key question is whether OSHMD can serve as a viable, more accessible tool for *in vivo* biological investigation, particularly of tumor vasculature. This case highlights OSHMDs potential to deliver clinically relevant insights, supporting broader use in research and practice.

## Conflicts of interest

None disclosed.
